# Assessing mild cognitive impairment using object‐location memory in immersive virtual environments

**DOI:** 10.1002/hipo.23458

**Published:** 2022-08-02

**Authors:** Andrea Castegnaro, David Howett, Adrienne Li, Elizabeth Harding, Dennis Chan, Neil Burgess, John King

**Affiliations:** ^1^ Institute of Cognitive Neuroscience University College London London UK; ^2^ School of Psychological Science University of Bristol Bristol UK; ^3^ Department of Psychology York University Toronto Ontario Canada; ^4^ Department of Clinical, Educational and Health Psychology University College London London UK

**Keywords:** Alzheimer's disease, entorhinal cortex, spatial cognition, virtual reality

## Abstract

Pathological changes in the medial temporal lobe (MTL) are found in the early stages of Alzheimer's disease (AD) and aging. The earliest pathological accumulation of tau colocalizes with the areas of the MTL involved in object processing as part of a wider anterolateral network. Here, we sought to assess the diagnostic potential of memory for object locations in iVR environments in individuals at high risk of AD dementia (amnestic mild cognitive impairment [aMCI] *n* = 23) as compared to age‐related cognitive decline. Consistent with our primary hypothesis that early AD would be associated with impaired object location, aMCI patients exhibited impaired spatial feature binding. Compared to both older (*n* = 24) and younger (*n* = 53) controls, aMCI patients, recalled object locations with significantly less accuracy (*p* < .001), with a trend toward an impaired identification of the object's correct context (*p* = .05). Importantly, these findings were not explained by deficits in object recognition (*p* = .6). These deficits differentiated aMCI from controls with greater accuracy (AUC = 0.89) than the standard neuropsychological tests. Within the aMCI group, 16 had CSF biomarkers indicative of their likely AD status (MCI+ *n* = 9 vs. MCI− *n* = 7). MCI+ showed lower accuracy in the object‐context association than MCI− (*p* = .03) suggesting a selective deficit in object‐context binding postulated to be associated with anterior‐temporal areas. MRI volumetric analysis across healthy older participants and aMCI revealed that test performance positively correlates with lateral entorhinal cortex volumes (*p* < .05) and hippocampus volumes (*p* < .01), consistent with their hypothesized role in binding contextual and spatial information with object identity. Our results indicate that tests relying on the anterolateral object processing stream, and in particular requiring successful binding of an object with spatial information, may aid detection of pre‐dementia AD due to the underlying early spread of tau pathology.

## 1 INTRODUCTION

The pathological accumulation of tau is associated with the spatial and episodic memory impairments that characterize both healthy aging (Crary et al., 2014; Hirni et al., 2016; Khan et al., 2014; Maass et al., 2019; Reagh et al., 2018) and Alzheimer's disease (AD) (Backman et al., 2004; Dubois et al., 2007; McKhann et al., 2011). The transneuronal propagation of tau begins in the parahippocampal gyrus, notably the trans‐ and lateral‐entorhinal cortex (EC), ahead of infiltrating the medial EC, wider medial temporal lobe (MTL) including hippocampus (HC) and the neocortex (Berron et al., 2021; Braak & Braak, 1997; Braak & del Tredici, 2011a; de Calignon et al., 2012). Unlike hippocampal degeneration, tau deposition in the EC/transentorhinal cortex appears to be specifically linked to AD, with a direct involvement in episodic memory decline even in normal aging, and in the absence of Aβ amyloid, another important AD marker (Adams et al., 2019; Maass et al., 2018). Given this initial involvement of the MTL development of behavioral tests sensitive to the hippocampal formation, dysfunction may facilitate the detection of preclinical AD.

The posterior medial–anterior temporal (PMAT) model (Kim et al., 2018; Ranganath & Ritchey, 2012; Ritchey et al., 2015) postulates two domain‐specific pathways in the MTL that converge in the EC. The anterior‐temporal system (including perirhinal cortex) processes object identity and content information, whereas, the posterior‐medial system (including the parahippocampus) is involved in spatial‐contextual information. This functional and anatomical division is maintained within the EC; the anterior‐lateral entorhinal cortex (alEC) and posterior‐medial entorhinal cortex are densely and reciprocally connected with the object and spatial processing streams, respectively (Maass et al., 2015; Reagh et al., 2018; Schröder et al., 2015). Such representations are subsequently bound downstream in the HC with temporal and emotional information forming the basis of episodic memory (Eichenbaum et al., 2012; Ekstrom & Ranganath, 2018; Ranganath, 2010).

The HC plays a role in cognitive mapping and episodic memory through the consolidation and long‐term storage of object details (Barense et al., 2007, 2011; Clarke et al., 2010), recognition of familiar objects in novel contexts (Cowell et al., 2010; Piterkin et al., 2008) and processing object‐location information (Bird & Burgess, 2008). Hippocampal recruitment is higher when object representations depend on an allocentric (object‐to‐object relations) framework, (Fidalgo & Martin, 2016; Hartley et al., 2007) or when objects are associated with landmarks or boundaries (Doeller et al., 2008).

While the HC is fundamental in the successful representation of object‐binding within contexts, emerging evidence suggests that such process might happen upstream in the hierarchy and could subserve the role of the HC (Knierim et al., 2014). For example, the perirhinal cortex has been proposed to encode object identity (Diana et al., 2007, 2012) by supporting a multimodal representation of features, (Taylor et al., 2006) necessary for fine‐grained discrimination of similar objects (Kivisaari et al., 2012). Subsequently, this information acts as an input to the alEC at the apex of the object‐processing stream. Rodent studies of the lateral EC (lEC) suggest that lEC lesions do not affect object identity recognition per se, (Deshmukh & Knierim, 2011; Wilson, Langston, et al., 2013; Wilson, Watanabe, et al., 2013) but appear to disrupt the process of identifying displacement of familiar objects, novel objects in familiar spatial contexts (van Cauter et al., 2013), or new object‐context associations (Chao et al., 2016). Additionally, lEC lesions in rodents selectively impair the ability to learn a spatial framework task (Kuruvilla & Ainge 2017). “Object” cells have been identified in the entorhinal cortices of both rodents (Tsao et al., 2013) and humans (Qasim et al., 2019) that fire selectively when approaching the location of an object regardless of whether the objects is present (Deshmukh & Knierim, 2011; Tsao et al., 2013; C. Wang et al., 2018). Taken together, this suggests that the lEC is vital in supporting memory “traces” that represent the conjunction of object identity, location, and spatial context (Hunsaker et al., 2013).

In humans, altered alEC activity has been associated with difficulties in differentiating familiar objects from decoys in older adults (Berron et al., 2018; Reagh et al., 2018; Wilson, Watanabe, et al., 2013). Importantly, the volumes of alEC, but not HC, have been associated with more accurate object‐location and intra‐object feature binding (Yeung et al., 2017, 2019). Object‐location binding deficits have been reported in older adults (Pertzov et al., 2012, 2015) and AD patients (Liang et al., 2016; Parra et al., 2009, 2010). In prodromal AD, manifested clinically as amnestic mild cognitive impairment (aMCI), patients exhibited deficits in a continuous distance error score in an object‐replacement task. Importantly, impairments were predicted more by the EC and parahippocampal gyrus volumetry than the hippocampal volume (Hampstead et al., 2018), indicating the diagnostic potential of tests probing the functions of these former regions. A recent study revealed that the volume of alEC, but not of HC, is associated with the ability of recollecting similar objects in aMCI indicating the emerging role of the anterior lateral regions in the object processing stream (Besson et al., 2020). In this context, performance on behavioral paradigms targeting EC processing, such as path integration, have demonstrated superior diagnostic differentiation between positive and negative AD biomarker status in MCI patients than traditional cognitive testing (Howett et al., 2019).

The current study reports on an object location task (OLT) to assess feature binding deficits in aMCI patients and older healthy controls using immersive virtual reality (iVR). This method benefits from relying on important sensory inputs necessary for spatial cognition, such as optic flow, proprioceptive, and vestibular feedback while maintaining full control over the visuals of the environment. The OLT comprises: (i) an object‐location recall subtask assessing binding between objects and their locations, (ii) an object recognition subtask assessing objects recollection using similar lures, and (iii) an object‐in‐context recognition subtask assessing the binding between the context and the object. Our primary hypothesis was that compared to age‐matched controls, aMCI patients will be impaired in all subtasks of the OLT. The secondary hypothesis was that OLT performance will differentiate between (i) MCI patients with AD positive cerebrospinal fluid biomarkers from MCI patients with negative biomarkers and (ii) older and young participants. It was anticipated that OLT performance will exhibit greater classification accuracy for the MCI stage than a comprehensive neuropsychological battery. MRI volumetric analyses were undertaken to further investigate the structure–function relationships in the MTL subregions of interest. Specifically, hypothesis was that the performance in the object location subtask would correlate with alEC and HC volumes, performance in the object recognition subtask with both alEC and perirhinal cortex volumes and performance in the object‐in‐context subtask would correlate with alEC volumes.

## 2 METHODS

### 2.1 Participants

Patients with amnestic MCI (aMCI, *n* = 23) were recruited at the Cambridge University Hospitals NHS Trust Mild Cognitive Impairment and Memory Clinics. MCI status was initially diagnosed by neurologists with criteria based on Petersen (2004). Amnestic classification was indicated on objective testing of episodic memory impairment and when the memory domain was the most affected.

16 patients underwent CSF biomarker studies (amyloid‐β1–42, total tau, and phosphorylated tau) as part of their clinical diagnostic workup. Biomarker studies were undertaken using ELISA assay kits (Innotest, Innogenetics; Shaw et al., 2009). Thresholds for positivity were set as CSF amyloid β 1–42 < 550 pg/ml, CSF total tau >375 pg/ml and with a CSF tau/amyloid ratio of >0.8 (Mulder et al., 2010). Patients with equivocal CSF results in one of these three cut‐offs were not included—for example, low amyloid but normal total tau. A sub‐sample of biomarker‐positive MCI (MCI+, *n* = 9) and biomarker‐negative (MCI−, *n* = 7) have been identified. The remaining seven patients with MCI did not undergo CSF studies.

Age‐matched healthy control participants (*n* = 24) were recruited from the Joint Dementia Research developed by the National Institute for Health Research (NIHR). Older healthy control participants were recruited from the online repository available through the Joint Dementia Research.

Exclusion criteria for the older groups were the presence of any major neurological or psychiatric disorder (Staples et al., 2019), a history of alcohol excess, head trauma or any mobility, or visual impairment which may compromise performance in iVR testing. A cohort of young (<40) healthy participants (*n* = 53), was recruited from the online participants pool UCL Sona. Exclusion criteria for the younger group included a history of mental disorders and visual impairments.

Ethical approval was granted by the NHS Cambridge South Research Ethics Committee (REC reference: 16/EE/0215) and the UCL Research Ethics Committee (ID number: SHaPS‐2018‐JK‐027). Both ethics were undertaken in line with the regulations outlined in the Declaration of Helsinki (WMA, 2013).

### 2.2 The object location task

The object location task (OLT) is administered using the HTC Vive iVR kit using a tracked walkable area of 4.0 × 4.0 m. The task was entirely developed using the Unity game engine (v2017.0.4f1) and consisted of three subtasks: (i) a spatial memory subtask where participants learned the locations of different objects in a cue‐rich environment and replaced them after a short‐time delay (~2 min), (ii) an object recognition subtask where participants choose whether a cued object was previously seen or not, and (iii) a context memory subtask where participants linked the previously seen objects to the environment the objects belonged.

**FIGURE 1:**
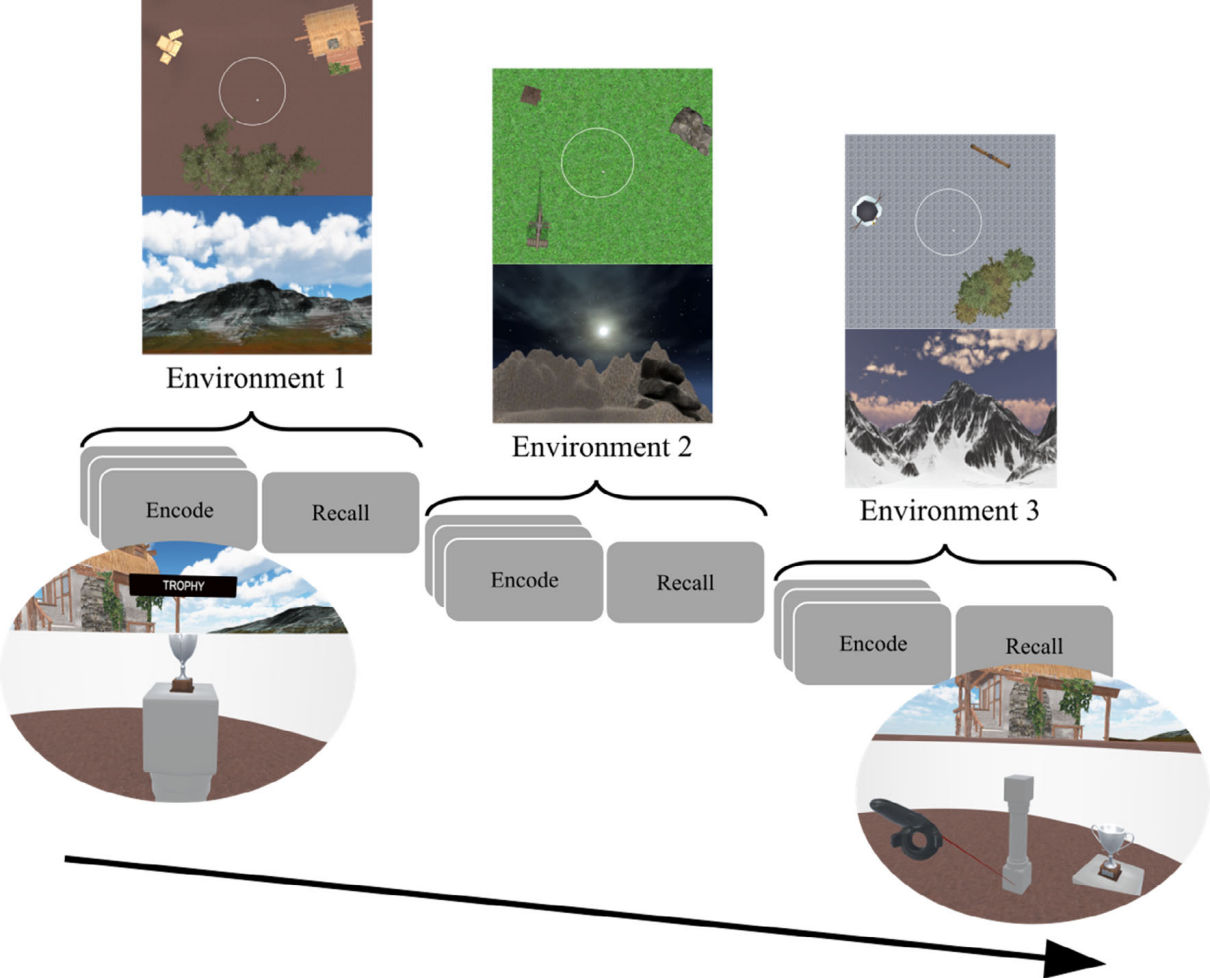
Object location memory subtask design. Design structure for the object location memory subtask. For each environment, participants underwent an encode and recall phase. During the encode phase participants walked to four different objects, one at a time (example in the view below the first encode box from the left). During the recall phase participants were asked to replace the previously seen objects by using the handheld controller which acted as a laser pointer (example in the view below the recall box). During the task participants visited three visually distinct environments featuring a 10 virtual meters circular colorless wall placed at the center of the virtual reality tracked area (one virtual meter corresponds to one real meter). Each environment had distinct distant landmarks (mountains with different textures and shape), near landmarks, light setting (daylight, night, and sunset), and sky texture

To facilitate the object‐context binding, visually distinct environments were created using distinct floor textures and landmarks (please refer to Figure 1). A circular boundary, placed outside of the iVR tracking area and with a radius of 10 m, was always present in the environment to aid participant's navigation and to replicate a previous study looking at hippocampal activity when learning object locations in the presence of boundary‐related information (Doeller et al., 2008).

Each participant completed a shorter version of the task to familiarize themselves with the virtual reality experience. The data collected from the practice was not further processed and thus excluded from any analysis. The total time required for testing each participant, including breaks, was around 50 min (2–5 min for set‐up, 15 min for practice, and 30 min for the full task). No participant reported fatigue or motion sickness following the virtual reality exposure and no participant dropped out from the experiment.

### 2.3 Object location memory

In the object location memory subtask, participants completed an “encoding” and “recall” phase (please refer to Figure 1 for the design of the subtask) in each of the three environments.

During encoding participants were required to memorize the locations of everyday objects while immersed in one of the virtual environments. Objects were sourced from a previous experimental paradigm investigating aging effects on short‐term memory (Hoefeijzers et al., 2017). For each environment, four different objects were presented on a gray pedestal in a pseudo‐random location and in a pseudo‐random sequential order, with only one object presented at any time. To encode each location, participants were asked to walk to the location of the pedestal. Upon reaching the pedestal location, the pedestal/object disappeared and the following item in the sequence appeared on a pedestal at another location. The component of active navigation by walking was a design choice made to enhance memory performance as previously demonstrated in a study assessing spatial cognition in virtual reality in older population (Sauzéon et al., 2016) and to mirror an experiment that identified “object” cells in humans in the EC (Qasim et al., 2019).

During the recalling phase participants were asked to remain stationary at the center of the environment. Objects were cued sequentially in a pseudo‐random order by presenting the object at the center of the field of view. In addition, after presentation, the cued object was always presented in the peripheral area of the participant's vision. The handheld controller acted as a laser pointer, with the tip presented as the gray pedestal, to help participants in replacing the location of the cued objects (please refer to Figure 1).

During encoding phase participants visited each object three times, whereas during recall they had only one chance to replace each object. Between encoding/recall phases and between different environments, participants were placed in a virtual “waiting” room devoid of any contextual cue.

An algorithm assigned four different random objects (from a pool of 28) to each of the three environments ensuring no object was presented in more than one environment and ensuring each object was located at least 1 m apart from the others.

### 2.4 Object recognition

In the object recognition subtask participants were placed in the virtual “waiting” room.

**FIGURE 2:**
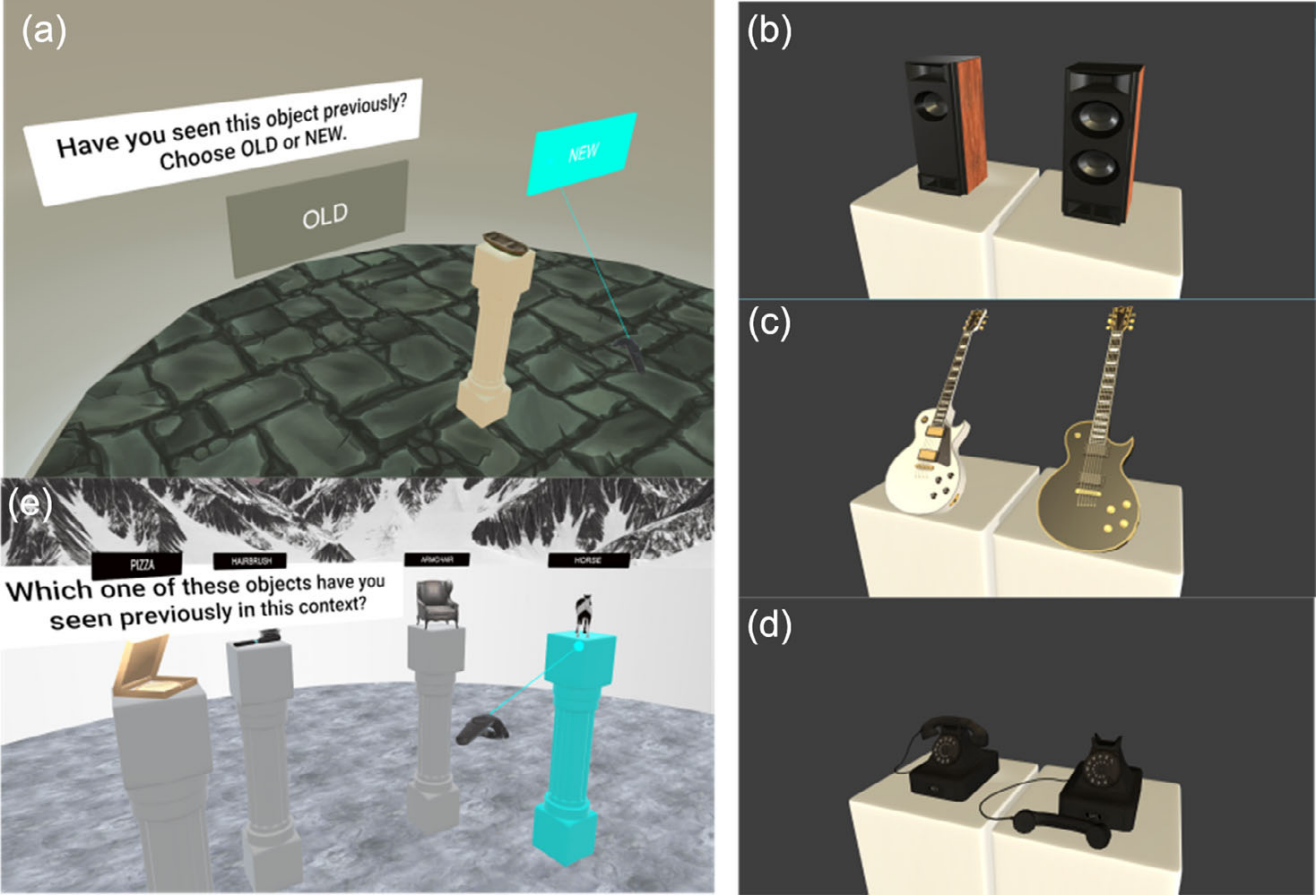
Object recognition and object‐in‐context subtasks. During the object recognition subtask, participants were kept in the “waiting” room devoid of any contextual cue (a). An object was placed on a pedestal in front of the participants along with two virtual cards labeled “Old”/“New.” Participants selected one of the cards using the handheld controller acting as a laser pointer depending on whether the object was previously seen in any of the environment (“Old”) or not (“New”). After the selection, participants were asked to give a four‐scale confidence rating to their choice with the following choices: “Certain”, “High Confidence,” “Low Confidence,” and “Guess”. “New” objects were created by pairing with “Old” objects and allowing one of the following changes: change in the number of a featured part (b), a texture change (c), and pose change (d). During the object‐in‐context subtask participants were placed back in one the previously visited environment and were shown four different objects at four different pedestals (e). Only one of the objects shown belonged to that environment. Participants made the selection using the handheld controller acting as a laser pointer

At the start of each trial, an object appeared in front of the participant on the gray pedestal together with two cards labeled as “Old” or “New” (Figure 2a). Using the handheld as a laser pointer, participants selected one of the cards choosing whether the object was previously seen in the environment (“Old”) or not (“New”). “Old” objects were paired to the “New” objects such that only one feature was changed from the original object, following one of three changes; (i) texture change, (ii) pose change, and (iii) change in the number of a particular feature of the object—for example, if showing flowers, the number of stems was different (Figure 2b–d). Participants completed 12 trials, with six “New” objects randomly chosen at the beginning of the subtask. Similar lures were a design choice instructed by the OLT pilot testing which revealed a ceiling effect on recognition performance in a few healthy older and aMCI participants when unrelated lures were used.

### 2.5 Object‐in‐context

In the object‐in‐context subtask, participants were asked to identify the environment previously associated with the objects (Figure 2e). After being virtually placed back in one of the environments, participants were shown four different objects on four pedestals. Each object had been previously seen during the encoding phase, but only one belonged to the current environment. Using the virtual pointer, participants could highlight the pedestal and confirm their choice. An algorithm chose foils from objects belonging to other environments avoiding the objects the participants selected during previous trials. Participants completed nine trials.

### 2.6 Outcome measures

The primary outcome for the object location memory subtask was the absolute distance error (ADE), in virtual meters, between the indicated and original object location. Lower displacement errors indicated higher accuracy and better performance on the task. The primary outcome of the object recognition subtask and the object‐in‐context memory was the percentage of correct choices.

Secondary outcome measures were the response times, that is, the time required for the participant to give an answer upon being prompted with the stimuli.

### 2.7 MRI acquisition

All older participants underwent MRI scanning on 32 channel Siemens 3T Prisma scanners based either at the MRC Cognition and Brain Sciences Unit, Cambridge, or at the Wolfson Brain Imaging Centre, Cambridge, with the same acquisition parameters used at the two scan sites. The scan protocol included whole brain 1 × 1 × 1 mm T1‐weighted MPRAGE (acquisition time 5 min 12 s, repetition time 2300 ms, echo time 2.96 ms) and high‐resolution 0.4 × 0.4 × 2 mm T2‐weighted scans through the hippocampal formation with scans aligned orthogonally to the long axis of the HC (acquisition time 8 min 11 s, repetition time 8020 ms, echo time 50 ms). D.H. collected and extracted the MRI structures. All the scans were acquired within a period of 3 months from the OLT task.

### 2.8 Comparator neuropsychological tests

A neuropsychological test battery was administered to the older participants recruited for the study. These tests have been chosen to provide a baseline comparison for the OLT. A list of the tests adopted can be found in Table 1. The neuropsychological battery test was performed within 1 week from the administration of the OLT.

**TABLE 1:** Demographics and neuropsychological tests

					Patients			
		Testing time (*m*)	Young controls (*n* = 53)	Healthy controls (*n* = 24)	MCI (*n* = 23)	Unknown (*n* = 7)	Negative (*n* = 7)	Positive (*n* = 9)
Age		‐	24.1 ± 3.0	68.8 ± 5.7*	71.7 ± 7.5n.s.	73.1 ± 8.3	67.2 ± 6.4	74.1 ± 7.0n.s.
Males (%)		‐	22(41)	8 (33)n.s.	13(57)n.s.	3 (43)	6 (67)	4 (57)n.s.
Years in education		‐	18.5 ± 2.7	15.5 ± 3.9n.s.	15.3 ± 3.4n.s.	16.0 ± 3.2	14.6 ± 4.2	15.4 ± 3.0n.s.
ACE‐R		15	‐	98.8 ± 2.5	85.9 ± 11.1*	90.9 ± 6.2	90.0 ± 7.3	78.9 ± 13.6n.s.
MMSE		10	‐	29.9 ± 0.3	26.5 ± 4.6 *	28.3 ± 1.8	28.7 ± 2.2	23.4 ± 5.9n.s.
NART		5	‐	6.2 ± 3.0	10.4 ± 8.3n.s.	10.1 ± 7.4	14.1 ± 10.9	7.8 ± 6.4n.s.
Rey complex figure	Copy	5	‐	36.0 ± 0.0	33.2 ± 3.9n.s.	33.3 ± 3.5	35.1 ± 1.2	31.7 ± 5.1n.s.
	Immediate	5	‐	22.9 ± 6.9	12.6 ± 10.0*	16.0 ± 10.5	14.4 ± 7.8	8.6 ± 10.7n.s.
	Delayed	5	‐	22.2 ± 7.1	12.1 ± 11.3n.s.	16.4 ± 11.6	14.1 ± 9.7	7.1 ± 11.4n.s.
FCSRT immediate	Free	5	‐	34.4 ± 5.1	20.2 ± 12.7*	26.4 ± 11.9	25.0 ± 8.8	11.7 ± 12.0*
	Total	5	‐	47.8 ± 0.5	40.0 ± 11.1n.s.	46.4 ± 3.7	44.7 ± 7.8	31.6 ± 12.0n.s.
FCSRT Delayed	Free	5	‐	13.4 ± 1.4	7.7 ± 6.0*	10.7 ± 5.6	8.9 ± 5.0	3.7 ± 5.2n.s.
	Total	5	‐	16.0 ± 0.0	14.3 ± 3.9n.s.	15.4 ± 1.5	15.0 ± 2.6	10.4 ± 4.4n.s.
Trail making (B)		10	‐	67.8 ± 21.1	135.1 ± 76.8*	112.0 ± 49.3	105.4 ± 32.3	176.1 ± 102.3n.s.
Digit symbol		5	‐	67.8 ± 13.1	46.8 ± 12.8n.s.	48.0 ± 16.2	49.7 ± 6.0	43.6 ± 14.5n.s.
4MT		10	‐	11.0 ± 1.9	8.2 ± 3.1*	10.3 ± 3.5	8.0 ± 3.0	6.8 ± 1.9n.s.

*Note*: This table summarizing the demographics of the different cohorts tested with the OLT and the results of the neuropsychological test battery for assessing aMCI and healthy older participants. Young controls and healthy controls were used to assess aging effect on the task performances. The MCI cohort was matched with the healthy controls in age and in years spent in education. Within the MCI cohort, a sub‐sample of patients were tested using the CSF biomarkers of Alzheimer's disease (CSF negative and CSF positive). A test battery was adopted to assess the aMCI and healthy older age‐matched controls. There were no age nor years in education differences between the CSF sub‐samples of the MCI. Composite scores were the Addenbrooke Cognitive Examination Revised (ACE‐R; Mathuranath et al., 2000) and the Mini Mental State Examination (MMSE; Folstein et al., 1975). Test abbreviations are as follows: National Adult Reading Test (NART; Nelson & Willison, 1991), Rey complex figure (RCF; Corwin & Bylsma, 1993), Free and Cued Selective Reminding Test (FCSRT; Buschke, 1984), Trial Making Test side B (TMT‐B; Reitan, 1958), Digit Symbol Substitution Test (DSST; Ryan & Lopez, 2001), Four Mountain Test (4MT; Hartley et al., 2007). Between group differences in each test performances were assessed between the healthy age matched controls and pooled MCI (including positive, negative, and unknown CSF status). A separate comparison was made between the CSF biomarkers positive and negative sub samples of the MCI. *p < .05 (Bonferroni adjusted to control for the number of comparisons).

### 2.9 Analysis

Demographic differences—namely, age at testing, years of education, and sex—between healthy controls and pooled MCI sample (MCI+, MCI, and MCI unknown) were assessed using an independent *t*‐test or chi‐square for categorical values. *t*‐Tests were substituted with the Wilcoxon–Mann–Whitney test when the assumption of normality for the data distribution or equal variances were not met. Demographic differences within MCI subtypes (MCI+, MCI−, and MCI unknown) were assessed using a one‐way ANOVA, or the Kruskal–Wallis test when the hypothesis of normality for the data distribution was not met. See Table 1 for a summary of demographic.

### 2.10 Neuropsychological tests

Differences in neuropsychological tests were assessed only in the older groups (healthy controls and MCI). Pooled MCI were compared against the older cohort and MCI+ were compared against MCI−.

### 2.11 Object location memory performance

Linear mixed effect models were used to assess the object location subtask performance measured as the absolute error distance (ADE) between the real and replaced location of the objects. Linear mixed effect modeling was the primary choice for addressing the within‐participant variability as each single trial was entered separately in the model and for addressing the nonrandomized design of the environments between participants. The model fitted (with slopes set to zero when the information was not present) is reported below:

$${\mathrm{DV}}_{\mathrm{ij}}=\beta_{0}+\beta_{1}{\text{Group}}_{j}+\beta_{2}{\mathrm{Age}}_{j}+\beta_{3}{\mathrm{Sex}}_{j}+\beta_{4}{\mathrm{Edu}}_{j}+\beta_{5}{\text{ACER}}_{j}+\beta_{6}{\text{NART}}_{j}+\;\beta_{7}{\mathrm{Env}}_{\mathrm{ij}}+\beta_{8}{\text{RetrievalTime}}_{\mathrm{ij}}+\beta_{9}\left({\mathrm{Age}}_{j}\times {\text{RetrievalTime}}_{\mathrm{ij}}\right)+\beta_{10}\left({\mathrm{Age}}_{j}\times {\mathrm{Sex}}_{\mathrm{ij}}\right)+U_{0j}+U_{1j}{\mathrm{Env}}_{\mathrm{ij}}+U_{2j}{\text{ObjectID}}_{\mathrm{ij}}+\;e_{\mathrm{ij}},$$

where DV_*ij*_ is the ADE for each trial *i* (1, …, 12) of participant *j*. *β*_0_ is the coefficient for the population mean, *β*_1_ is the coefficient for the categorical variable indicating group or diagnostic status of participant j depending on the hypothesis. Age (*β*_2_), sex (*β*_3_), years in education (*β*_4_), ACE‐R (*β*_5_), and NART (*β*_6_) have been included as additional fixed effect to control for participant *j* differences in cognitive status and premorbid IQ, respectively. *β*_7_ is a fixed effect to model the three different environments and *U*_1*j*_ is a random slope allowed for each participant to control for performance differences in each environment. *β*_8_ is a fixed effect to model the error based on the retrieval time, or the search of cues within each environment. *β*_9_ and *β*_10_ are slopes for the interaction terms between the age and retrieval time and between age and sex. *U*_0*j*_ is the random intercept for each participant and *U*_2*j*_ is a random slope modeling the randomization of the objects. Finally, the term *e*_*ij*_ is the trial‐level error for each trial *i* of participant *j*. Final models were informed by a mixture of apriori hypothesis and important covariates found in literature in aging and dementia (sex, education). Random effects controlled for differences in performance in the environments and the types of objects presented. Three different models were run to independently investigate the three research questions, specifically: the effect of MCI status between the healthy age‐matched older control and MCI group, the effect of CSF status between the MCI+ and MCI−, and the effect of aging between young and older healthy participants. Denominator degrees of freedom were reduced using the Satterthwaite approximation.

### 2.12 Detection of location binding errors

Location binding errors, where a cued object is reported in place of another object, are reported in aging (Muffato et al., 2019; Pertzov et al., 2015) and patients affected with aMCI (Hampstead et al., 2018) and AD dementia (Liang et al., 2016). For this reason, location binding errors are of interest as they could be confounding in the reported distance associated with a cued object (Bays, 2016). In this study, we assessed the aposteriori hypothesis that the frequency of location binding errors will be greater in MCI. To assess the location binding errors, a heuristic detection algorithm based on the geometry of each configuration of objects has been implemented (see supplementary information S1 for more information). The algorithm identified whether an object was re‐located in the proximity of another object in the same configuration. Hypothesis testing has been performed by fitting a generalized linear model with a Poisson distribution (Poisson regression). The model fitted is reported below:

$${\mathrm{DV}}_{\mathrm{ij}}=\;\beta_{0}+\;\beta_{1}{\text{Group}}_{j}+\beta_{2}{\mathrm{Age}}_{j}+\beta_{3}{\mathrm{Sex}}_{j}+\beta_{4}{\mathrm{Edu}}_{j}+\beta_{5}{\text{ACER}}_{j}+\beta_{6}{\text{NART}}_{j}+\beta_{7}\left({\mathrm{Age}}_{j}\times {\mathrm{Sex}}_{\mathrm{ij}}\right)+U_{0j}+\;e_{\mathrm{ij}},$$

where DV_*ij*_ is the number of misplaced locations or retrieval failure for each configuration *i* (1, …, 3) of participant *j*.

### 2.13 Recognition and object‐in‐context memory

Signal detection theory was applied to quantify the decision‐making process used by participants in discriminating objects in the memory recognition subtask (Mahoney et al., 2015). *D*′ mean differences between groups have been assessed using an ANOVA covarying for age, sex, and years in education. In the object‐in‐context subtask, differences in the percentages of correct choices between young, older healthy, and pooled MCI have been assessed using an ANOVA covarying for age, sex, and years in education. A general linear model was used to assess the CSF status on the object‐in‐context performance between MCI+ and MCI− featuring the age, sex, and years in education, NART and ACE‐R as additional fixed effects. Additionally, given the small sample size and the data quantization leading to a deviation from normality, we assessed the median differences between young, healthy older, and pooled MCI using a Kruskal–Wallis, and the median difference between the MCI+ and MCI− using a Mann–Whitney–Wilcoxon test (see supplementary material S1).

### 2.14 Receiver operating characteristics

The OLT's ability to differentiate MCI from healthy older adults was compared to the reference neuropsychological test battery's classification accuracy.

To extract the specificity and sensitivity of the classification, a logistic classifier has been trained using maximum likelihood principles. Additional parameters controlled for age, sex, and years in education. A 10k‐fold cross‐validation was used to avoid overfitting of the models. Posterior probabilities of the model were used to generate the receiver operating characteristics (ROC) and to calculate the area under the curve (AUC). AUC confidence intervals have been generated by bootstrapping with 1000 repetitions. Additionally, to systematically compare the OLT performance with the neuropsychological tests, a linear logistic model has been obtained using a stepwise elimination from a full model where the OLT subtasks performances and all the comparator neuropsychological test were inserted. Neuropsychological tests that did not show any statistical difference between groups were excluded and the MMSE was excluded given the overlap with the ACE‐R. For each step, the threshold for the chi‐square test measuring the deviance between the two models with and without the term under test was set to 0.1. The final model has been used to calculate the ROC and AUC as described above. Insufficient sample sizes for the aMCI with CSF biomarkers prohibited examining the classification accuracy for the CSF effect.

### 2.15 MRI volumetric analysis

Selected regions of interest (ROI) were the EC, alEC, HC, and perirhinal cortex. EC, alEC and perirhinal cortex were manually segmented on coronal slices of high resolution T2‐weighted 3T MRI scans using ITK‐SNAP (Yushkevich et al., 2006). Perirhinal cortex was identified in the Brodmann area 35 (BA35). The complete manual segmentation of the EC and BA35 was adapted from the protocol outlined in Berron et al. (2017) that aims to accommodate the anatomical variability of the collateral sulcus in this region (Ding & van Hoesen, 2015). Segmentation of the anterolateral subdivision of the EC were derived and adapted from Maass et al. (2015) and were described in detail in Howett et al. (2019).

Owing to their evidenced role in memory recollection and spatial processing, volumetric estimates of the HC (Bird & Burgess, 2008), precuneus (Weniger et al., 2011), inferior parietal (Maguire, Frith, et al., 1998; Weniger et al., 2011) and the isthmus cingulate cortices (Auger et al., 2012; Marchette et al., 2014; Uncapher et al., 2006) were segmented using the Desikian–Killiany atlas from Freesurfer 6.0 (Fischl et al., 2002; Iglesias et al., 2015). The isthmus cingulate cortex mask was used as a proxy measure of the retrosplenial cortex (does not include retrosplenial's agranular BA 30).

All segmentations were manually inspected to exclude cysts, CSF, and meninges; all volumetric measurements were averaged between hemispheres and normalized to estimated intracranial volume.

One‐way MANCOVA was used to examine the effect of ROIs across all of the OLT subtasks covarying for group, age, sex, and years in education across all older participants. Dependent variables were specified as the ADE in the object location memory subtask, and as the percentage of correctly identified objects in both the object recognition, and the object‐in‐context subtasks. Predictor variables included the whole EC, alEC, hippocampal, and BA35 volumes which were all mean centered.

To assess the association between ROI volumes and the three different OLT subtasks separately, three linear models with age, sex, years in education, and diagnostic status as additional fixed effects were run. The models also included the parietal regions to control for their involvement in spatial‐contextual memory.

## 3 RESULTS

### 3.1 Demographics and neuropsychological testing

Table 1 reports the demographics of the cohorts tested with the OLT and the results of the neuropsychological tests adopted for the older and aMCI participants. There were no differences in age, years in education, or sex between the healthy older controls and the pooled MCI cohort or between the MCI+ and MCI−, and no differences in sex or education were observed between young and older controls. The healthy older controls performed significantly better than the pooled MCI cohort in several tests (*p* < .002), for details see Table 1. Between the MCI− and MCI+ subgroups only the free and cued selective reminding test (free immediate recall) showed significant difference with worse performances in the positive group (*p* < .002).

### 3.2 Object location memory accuracy

**FIGURE 3:**
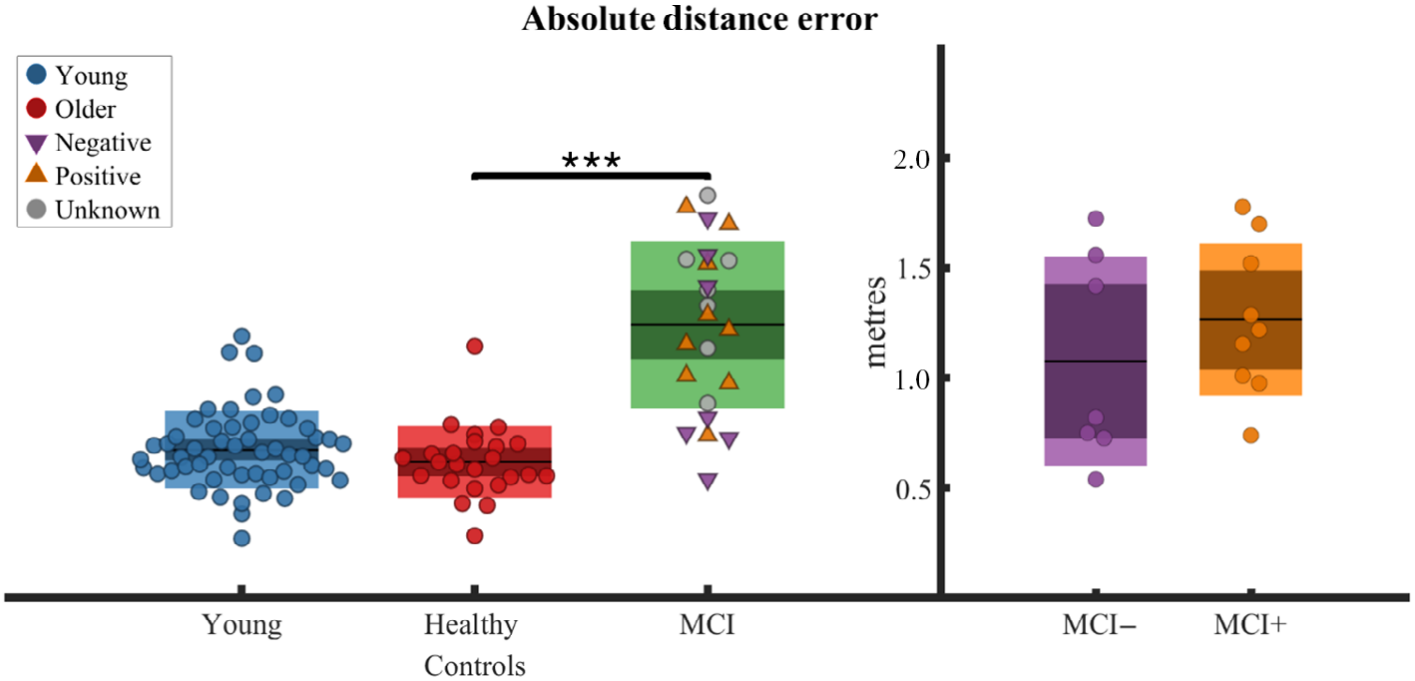
Object replacement accuracy. Absolute distance error per participant between veridical object locations and participant's responses averaged across trials reported for different groups. On the left panel groups presented are young, healthy older age‐matched controls, and pooled MCI (containing positive, negative, and unknown CSF status); on the right panel groups presented are MCI with negative/positive CSF status. Boxplots report the group mean as the thick black horizontal line, standard error of the mean as the darker area, and confidence interval as the external limits of the boxes. Significance bar indicates the main effect of MCI status on the absolute distance error found in the linear mixed effect model run across older age‐matched healthy controls and MCI and where each trial was inserted as a separate observation (***p < .001)

A significant main effect for MCI status was found on the AED (*β*_1_ = .64, CI = 0.33–1.01, *t*[1,50] = 3.4, *p* < .001, Figure 3a). In particular, the pooled MCI cohort exhibited larger ADE than the older healthy cohort by an estimated 0.51 ± 0.14 m.

No main effect of CSF status was found in ADE between MCI+ and MCI− patients. No main effect of aging was found in ADE between healthy older controls and young controls.

### 3.3 Location binding error frequency

**FIGURE 4:**
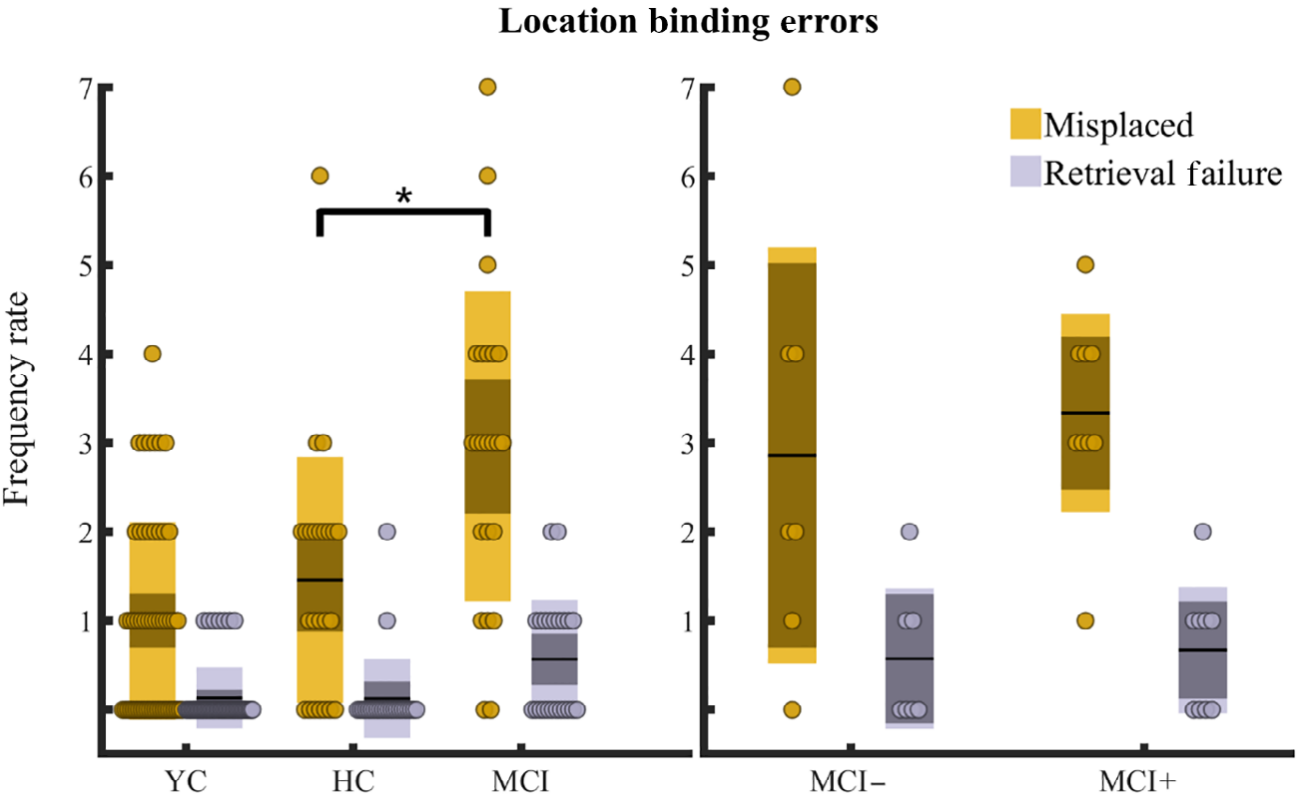
Binding errors frequency rate. Frequencies of the detected location binding errors were summed for each participant and reported for different groups. On the left panel groups presented are young, older age‐matched healthy controls (HC), and pooled MCI (positive, negative, and unknown CSF status). On the right panel groups presented are MCI with negative/positive CSF status. A binding error occurs when a participant replaced an object A in proximity of the veridical location of an object B in the same configuration. A heuristic in‐house algorithm (see Supplementary information S1) was developed to detect binding errors. When the algorithm detected at least three misplaced objects in a configuration, a retrieval error was registered. Yellow and gray circles indicate the sum of detected binding errors and retrieval errors, respectively, per participant. Boxplots report the group mean as the thick black horizontal line, standard error of the mean as the darker area, and confidence interval as the external limits of the boxes. Significance bar indicates the main effect of MCI status on the binding error count found in a generalized linear model run across older age‐matched healthy controls and MCI and where each configuration was inserted as a separate observation (*p < .05)

A significant effect of MCI status was found on the frequency of object‐location binding errors (*β*_1_ = .65, CI = 0.16–1.22, *z* = 4.68, *p* = .03; Figure 4a). The pooled MCI cohort exhibited a mean frequency increase of 0.50 ± 0.45 compared to older healthy controls. Interestingly, sex was also a significant fixed effect in the model (*β*_3_ = −.52, CI = −0.95 to −0.09, *z* = −2.42, *p* = .02) with females exhibiting fewer location‐binding errors than males across the older participants with a mean frequency decrease of 0.44 ± 0.82. No other effects were found when assessing the CSF status or the aging status in the location binding errors.

### 3.4 Object recognition performance

**FIGURE 5:**
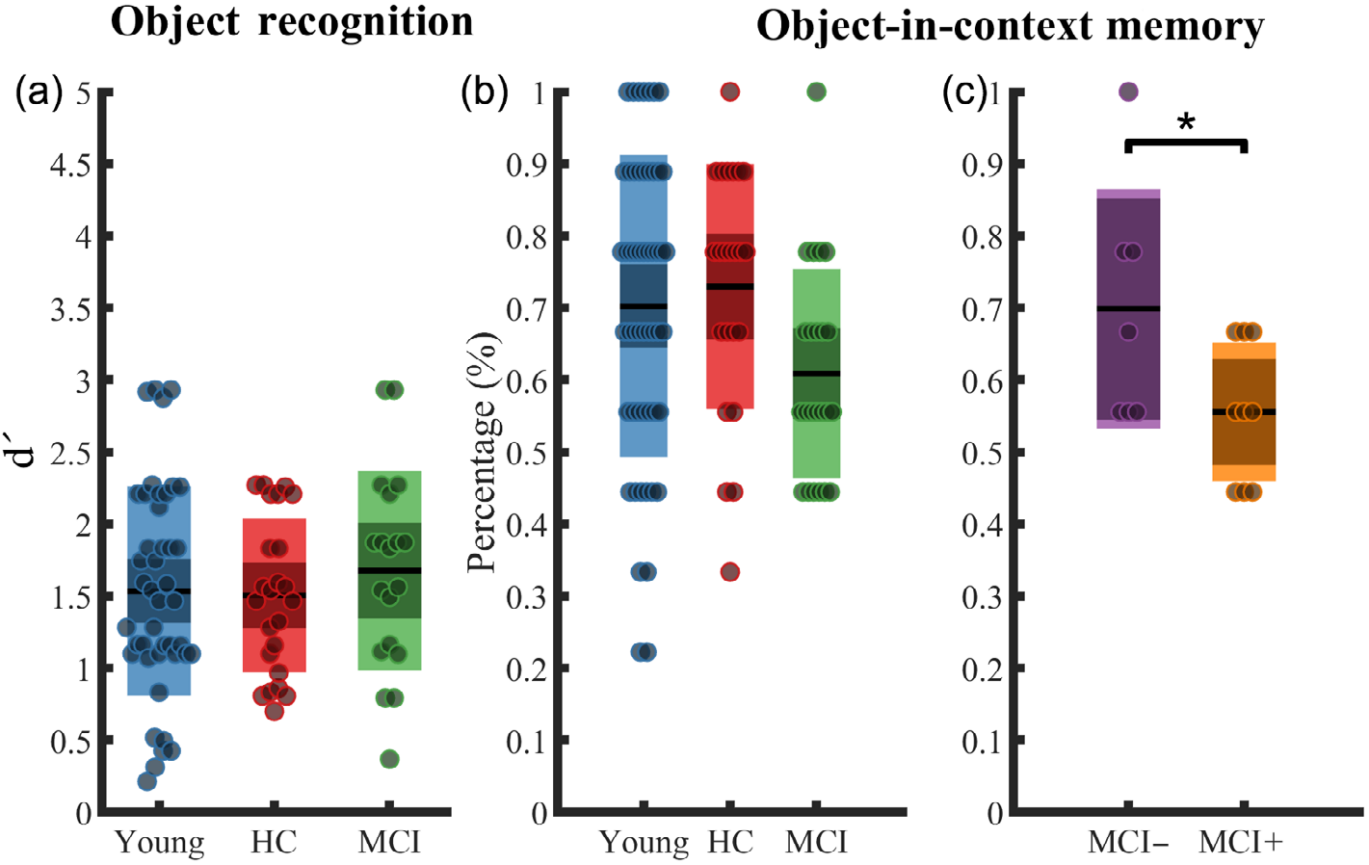
Object recognition and object‐in‐context memory performances. (a) Object recognition subtask performance. (b–c) Percentage of correct answers in the object‐in‐context memory subtask. Groups presented are young, older healthy age‐matched older controls (HC) and pooled MCI (positive, negative, and unknown) in (a, b), and MCI with negative/positive CSF status in (c). Mean is reported as a thick black horizontal line, standard error of the mean as the darker area, and confidence interval as the external limits of the boxes. Significance bar indicates the main effect of CSF status on the percentage of correct responses in the object‐in‐context subtask in a general linear model run across MCI+ and MCI−, and where each percentage score was inserted as a separate observation (*p < .05)

In the object recognition subtask, each group performed equally well in terms of overall accuracy (*F*[2,94] = 0.51, *p* = .6; Figure 5a).

### 3.5 Object‐in‐context memory performance

All groups performed above the chance (young, *m* = .70, *p* < .001; older control, *m* = .73, *p* < .001; MCI pooled, *m* = .61, *p* < .001). No main effect of MCI status was observed when comparing the percentage of correct answers in the object‐in‐context subtask between the healthy old controls and MCI patients (*F*[2,94] = 3.95, *p* = .05; Figure 5b). Interestingly, a significant main effect of CSF status was found in the percentage of correct responses between MCI+ (*m* = .56 ± .09) and MCI− (*m* = .70 ± .16) (*β*_1_ = −.18, CI = −0.32 to −0.04, *t*[1,10] = −2.6, *p* = .03; Figure 5c).

### 3.6 MCI classification

**FIGURE 6:**
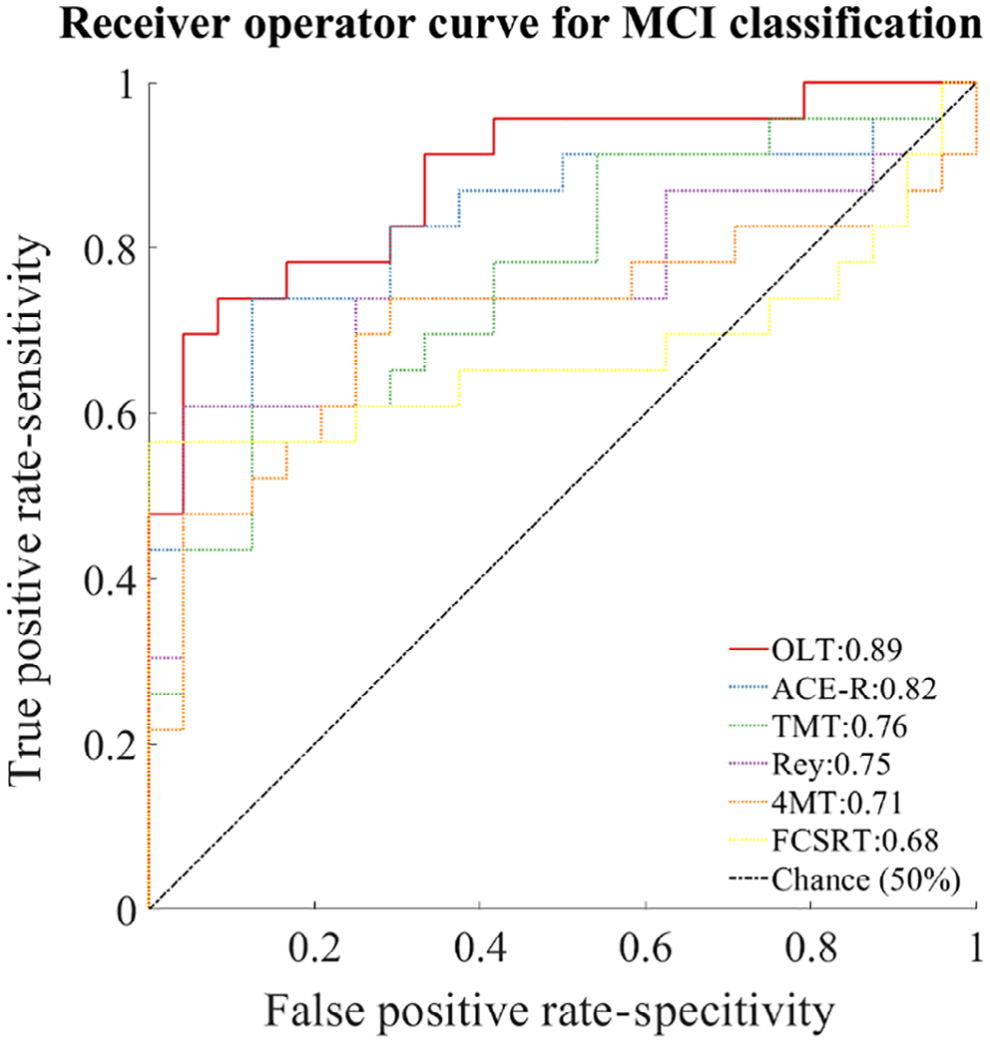
Receiver operating characteristics (ROC) curve. A logistic classifier has been trained to assess the ability of the neuropsychological tests in determining the MCI status against the healthy age‐matched controls. Pooled MCI (including patients with positive, negative, and unknown CSF biomarkers status) have been used for training the classifier. Only neuropsychological tests with performances that have been found statistically different between the two groups have been used for training the classifier. The classifier for the OLT has been trained with the performance from the object replacement subtask. The classifier for free and cued selective reminding test has been trained with the performance from the free immediate recall and the delayed free recall. The classifier for the Rey–Osterrieth figure recall test has been trained with the performance from the immediate recall only. ACE‐R, Addenbrooke cognitive examination‐revised; TMT, trail making test part B; 4MT, four mountain test. Chance level is represented by black dash‐dot line

Performance in the object replacement subtask (ADE) has been used to assess the OLT's ability to differentiate pooled MCI from older healthy controls. The OLT exhibited a classification performance with an AUC of 0.89 (CI = 0.72–0.95; Figure 6). Classification accuracy was lower for all the comparator neuropsychological tests: ACE‐R was associated with an AUC = 0.82 (CI = 0.71–0.94), trail making test part B [(AUC = 0.76; CI = 0.62–0.89)], Rey–Osterrieth complex figure test [(AUC = 0.75; CI = 0.56–0.86)], four mountains test (AUC = 0.71, CI = 0.51–0.84), free and cued selective reminding test (AUC = 0.68, CI = 0.47–0.83). A logistic classifier obtained by a stepwise elimination process revealed that OLT object recognition (*χ* ^2^ = 0.25, *p* = .78), trail making test part B (*χ* ^2^ = 0.20, *p* = .65), Rey–Osterrieth complex figure test (*χ* ^*2*^ = 0.32, *p* = .57), four mountains test (*χ* ^2^ = 1.20, *p* = .27), and the delayed free and cued selective reminding test (*χ* ^2^ = 2.40, *p* = .12) could be removed from the model. The obtained logistic model featuring OLT location memory subtask (*z* = 2.31, *p* = .02), ACE‐R (*z* = −2.02, *p* = .05), OLT object‐in‐context subtask (*z* = 1.89, *p* = .06) and immediate free and cued selective reminding subtask (*z* = −1.59, *p* = .11) yielded an AUC = 0.98 (CI = 0.94–0.99).

### 3.7 MRI volumetry and association with OLT performance

A multivariate analysis examined the relationship between the ROIs (alEC, HC, and BA35 encompassing perirhinal cortex) and the OLT performance across healthy controls and MCI participants. OLT performances were measured as the ADE in object location memory subtask, percentage of correct choices in the object recognition, and percentage of correct choices in object‐in‐context subtasks. Multivariate analysis revealed that OLT performance across subtasks was predicted by the alEC (PT = 0.31, *F*[3,33] = 4.86, *p* < .05) and hippocampal (PT = 0.33, *F*[3,33] = 5.35, *p* < .01) volumes.

**FIGURE 7:**
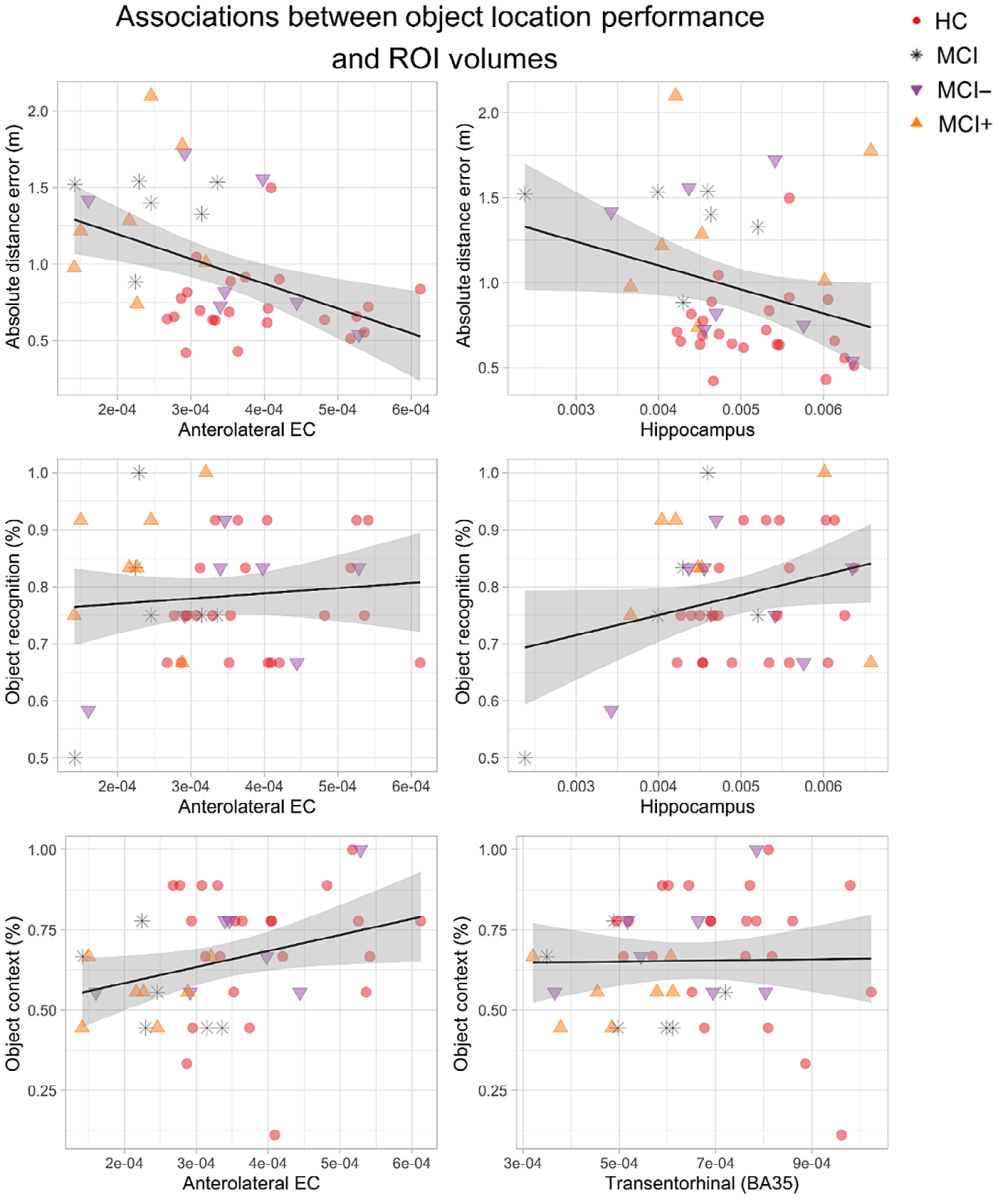
Associations between object location subtasks performance and regions of interest volumetry. Included graphs reflect the a‐priori selected ROIs, except for the association between object recognition and the hippocampus which was a far stronger predicted of object recognition than the hypothesized perirhinal (BA35) volume. In each graph the main outcome of the subtask is reported alongside the *y* axis while the *x* axis represents the extracted volumes corrected by estimated intracranial volumes. The black line reported is the result of a simple regression between the two reported quantities without controlling for age, sex, years in education, and diagnostic status, thus the slope might not capture the results of the multilevel modeling adopted for the volumetric analysis reported in Table 2. In line with our hypothesis, anterolateral EC volumes is associated with object‐in‐context performance and approach significance in the location memory subtask. The hippocampus, but not the entorhinal or perirhinal volumes, were predictive of object recognition performance

Looking at the OLT performance separately, larger hippocampal volumes were associated with more correct responses in the object recognition subtask (*t*[1,31] = 3.65, *p* < .001, *R* ^2^ = .36; Table 2), whereas the proportion of correct responses in the object‐context association subtask was associated with larger alEC (*t*[1,31] = 3.96, *p* < .001, *R* ^2^ = .48; Table 2). No volume was associated with the ADE in the object replacement subtask. Figure 7 reports the single associations between OLT subtasks performances and ROI.

**TABLE 2:** Associations between object location subtasks performance and volumes

	Object location memory			Object recognition			Object‐in‐context		
	*β*	SE	*p*	*β*	SE	*p*	*β*	SE	*p*
Entorhinal cortex	.23	.16	.17	−.10	.06	.09	−.20	.09	.03
Anterolateral EC	−.31	.14	.05	.06	.05	.20	.27	.07	<.001
BA35	.01	.08	.92	−.01	.03	.69	−.08	.05	.10
Hippocampus	.02	.10	.87	.39	.03	<.001	−.03	.05	.62
Isthmus cingulate	−.10	.08	.21	−.02	.03	.34	.01	.04	.90
Inf. parietal cortex	−.03	.08	.70	.04	.03	.15	.02	.04	.71
Precuneus	.01	.09	.90	−.03	.03	.25	.05	.05	.30
	*F*(11,31) = 4.68, *p* < .001, *R* ^2^ = .50			*F*(11,31) = 1.87, *p* = .08, *R* ^2^ = .36			*F*(11,31) = 3.53, *p* < .01, *R* ^2^ = .48		

*Note*: Volumes associations with performances of each object location subtask. Three linear models were used to assess each of the subtasks separately across pooled MCI and the healthy older control group with volumes averaged across hemispheres and normalized to estimated intracranial volume. Dependent variables were specified as absolute distance errors in the object location memory subtask, and the percentage of objects correctly identified in both the object recognition and object‐in‐context subtask. Regions of interest are anterolateral enthorinal cortex, perirhinal cortex (BA35), and hippocampus. Additional volumes as fixed effects are the entorhinal cortex (as a proxy for posterior‐medial region), the isthmus cingulate (as a proxy for the retrosplenial cortex), the inferior parietal cortex, and the precuneus owing to their role in spatial cognition. All models were adjusted for age, sex and years in education as well as diagnostic group to control for volumetric differences. Slopes, standard error and *p* values are reported for each volume inserted in the model. Bottom row represents the goodness of fit or the variability in the dependent model explained by each model.

## 4 DISCUSSION

Using a novel iVR OLT dependent on MTL function, this study demonstrated that patients with aMCI exhibited deficits in binding object identity with environmental location. In line with our primary hypothesis, impaired performance on the object replacement subtask successfully differentiated aMCI patients from age‐matched healthy controls with greater accuracy than a clinical battery of neuropsychological tests. However, the aMCI group's performance in object recognition and object‐context associations was not sufficient to accurately differentiate them from healthy age‐matched controls. These results suggest that the recall of object locations in a spatially rich environments is a selective deficit in aMCI. Of particular note is that object‐context association accuracy was significantly lower in aMCI patients with positive CSF AD biomarker status compared to their CSF negative counterparts, consistent with the former group having greater pathological changes in the MTL. As such, a task requiring remembering the configuration of different objects in specific contexts may have a significant diagnostic value, given the evidenced role of the alEC and hippocampal formation in binding objects related information and the vulnerability of this region to tau pathology in the earliest stages of AD.

### 4.1 Object location memory accuracy

The larger ADEs exhibited by the aMCI group compared to the healthy older control group is consistent with object‐location learning deficits found in discrete (Kessels et al., 2010) and continuous measure tasks (Hampstead et al., 2018; Sapkota et al., 2017).

Contrary to our hypothesis, object replacement was not affected by either aging or CSF evidence of AD pathology. This finding is surprising given that object placement deficits are present in early AD dementia (Liang et al., 2016) and can successfully differentiate MCI from AD staging (Kessels et al., 2010; Parra et al., 2009). In addition, during encoding participants were required to actively walk while learning the objects of the environment, thus involving a successful integration of self‐motion information, a function altered in aMCI due to AD (Howett et al., 2019; Kunz et al., 2015; Lithfous et al., 2013) and in healthy older people (Stangl et al., 2018).

One possible explanation for the null finding is that participants may rely on egocentric wayfinding strategies to remember the location of the objects (Chersi & Burgess, 2015; Goodroe et al., 2018), involving striatal areas that are affected only in the later AD stages (Berron et al., 2021; Braak & Braak, 1997; Braak & del Tredici, 2011b). This explanation is congruent with the preference for egocentric rather than allocentric navigational strategy found in aging and aMCI (Parizkova et al., 2018; Rodgers et al., 2012; Ruotolo et al., 2016) and with the observed attenuation of deficits when task demands a switch from egocentric to allocentric strategy (Ruggiero et al., 2017).

Our a posteriori hypothesis confirmed that higher location binding errors were observed in the aMCI as compared to controls. Misbinding errors are not explained by deficits in object identity or object location; all participants performed above chance in the object recognition memory subtask and our detection algorithm controlled for object proximity and the veridical position of all objects in a given configuration (see Supplementary information S1). Taken together, the aMCI deficits observed in the object location subtask are likely the result of errors in the binding of object identity with location. Collectively, these results are in line with the PMAT model, proposing a functional subdivision of the EC (Ranganath & Ritchey, 2012; Ritchey et al., 2015) and the evidenced role of the alEC as a crucial structure in supporting object trace memory in rodents and humans (Deshmukh & Knierim, 2011; Qasim et al., 2019; Reagh & Yassa, 2014; Tsao et al., 2013; Wang et al., 2018; Yeung et al., 2019). Furthermore, the analysis of location binding errors revealed a sex difference where females displayed less errors than males across the older participants. Sex differences are starting to emerge as a clinical difference in AD (see Dubal, 2020 for a review). Notably, females display a more preserved brain structure under similar levels of tau propagation (Ossenkoppele et al., 2020), suggesting greater levels of brain reserve (Digma et al., 2020), potentially explaining the result reported in this study. However, the result should be taken cautiously as similar trends were not find in the main outcome of the object location memory subtask or in the other subtasks of the study, and while reminding the importance to control for sex in AD, further investigation is required.

In line with our hypothesis, performance on the object location memory subtask differentiated aMCI from healthy age‐matched controls with higher accuracy than standard neuropsychology tests used in clinical assessment. Additionally, our analysis was strengthened by using a stepwise logistic classifier to select only the significant predictors from a full model containing all the OLT subtasks and the comparator neuropsychological tests adopted. The final logistic classifier featured the OLT location memory, ACE‐R, OLT object‐in‐context subtask, and FCSRT as the best model to predict the MCI status, however, only the OLT location memory had a coefficient statistically different from zero indicating the OLT location memory subtask as the best predictor for the MCI status. The classification accuracy was also higher than studies employing a 2D object‐replacement task (Wang et al., 2013) highlighting the clinical potential of iVR technology for clinical assessment.

### 4.2 Object recognition performance

Contrary to our hypothesis, no effects of MCI status, underlying pathological condition indexed by the CSF biomarker, or aging were observed in the object‐recognition subtask. This result is surprising given the anterior‐temporal demands of processing object identities (Berron et al., 2018; Diana et al., 2007) and its reported deficits in aMCI (Bennett et al., 2007; Stark et al., 2013; Tran et al., 2021) and preclinical AD (Gaynor et al., 2019). Recent research demonstrates that impaired mnemonic discrimination is predicted by increased CSF tau levels in cognitively unimpaired older adults (Berron et al., 2019; Düzel et al., 2018) and is present in preclinical AD (Trelle et al., 2021). Importantly, tau deposition in anterior‐temporal networks is associated with deficits in object discrimination (Maass et al., 2019) contrasting with the results of object recognition subtask of the present study. The null results may be due to a limited quantity of stimuli presented to each participant in the object recognition memory subtask (approximately 20% of the number of stimuli used in object recognition evaluations which found deficits in aMCI; see Bennett et al., 2007; Liang et al., 2016). Despite the limited number of stimuli, no ceiling/floor effects were observed, with groups performing above chance.

### 4.3 Object‐in‐context memory performance

No main effect of MCI status was observed in the performance of the object‐in‐context subtask between the healthy age‐matched older controls and the pooled MCI. However, when looking at the MCI subgroups with biomarkers, CSF status was a main effect in the object‐in‐context accuracy revealing that MCI− performed better than MCI+ counterparts. To our best knowledge, this is the first evidence that the binding between object identity and its context can be associated with underlying pathology in aMCI. Emerging evidence suggests that the encoding of contextual information depends on the anterior‐temporal network. For example, the rodent lEC has been demonstrated to support the integration of spatial and contextual information (Chao et al., 2016; Keene et al., 2016; van Cauter et al., 2013; Wilson, Langston, et al., 2013), especially in tasks where the context plays a role in the reward assignment (Keene et al., 2016). In humans, tau pathology in antero‐temporal networks generally affects activity during context discrimination (Maass et al., 2019), however when assessing object and context recognition separately, tau burden in alEC selectively affects object rather than context recognition performance (Berron et al., 2018, 2019). Considering the findings from the memory recognition subtask, our result suggests the possibility that binding of features in memory might be affected prior to the recognition of single features. This possibility is congruent with the alEC being a convergent point of the anterior‐temporal networks and supporting the representation of objects within contexts that subserve the HC to form the base of episodic memory (Maass et al., 2015). While we did not have the access to the specific locations of tau concentration in the patient cohorts, future studies should look at the possibility that tau presence in anterior temporal networks, particularly alEC, might manifest deficits in encoding objects relative to their contexts rather than deficits in remembering the single elements.

### 4.4 Volume associations with performance

Multivariate analyses revealed that bilateral alEC and HC volumes were associated with performances across the OLT. This result is in line with the evidence that alEC plays a role in processing object information within environments as demonstrated by lesion studies (Keene et al., 2016; Kuruvilla et al., 2020; Wilson, Langston, et al., 2013), functional imaging in humans (Berron et al., 2018, 2021; Maass et al., 2019) and furtherly supported by the direct recordings of a class of cells spatially tuned to retain a trace of previously learned objects (Qasim et al., 2019; Tsao et al., 2013; Wang et al., 2018). Whereas, previous research observed a significant association between the reduced volume of EC and impaired performance on the continuous measures of location memory in aMCI patients (Hampstead et al., 2018), our results were only trending in this direction. It is worth noting that other research proposed that changes in the activity of both the alEC and HC may be associated with the behavioral impairment in location discrimination in older people (Reagh et al., 2018). Given the low statistical power in our study bound to the small number of patients, it remains yet to be determined whether it was the underlying neuropathology in the alEC, HC, or both, that was driving the behavioral impairment in aMCI as compared to the healthy elderly control observed in our study.

Although we did not observe a significant association between the ROI and the behavioral performance on our task, our object location memory subtask still exhibited superior classification accuracy in differentiating between MCI patients and age‐matched controls as compared to the standard neuropsychological tests. One explanation for this greater discriminatory potential is that the OLT presents higher demands in the executive and spatial domains, as it requires orientational, perceptual, motor, and proprioceptive precision. Such a broad range of demands, alongside spatial memory and attention network requirements may be sensitive to synaptic dysfunction. Critically, the dysfunction at the level of signal transfer between synapses was proposed to precede brain atrophy by several years, as demonstrated for parietal and frontal regions (Broadhouse et al., 2021; Gili et al., 2011). This explanation is reinforced by a recent evidence on the link between synaptic dysfunction in the EC and memory decline in nonhuman primates (Long et al., 2020).

Previously, the volumes of HC, but not the perirhinal cortex, were observed to be associated with the performance in object recognition in older adults (Broadbent et al., 2010; Cohen et al., 2013). Indeed, our results are consistent with the previous studies proposing that the HC plays a role in long‐term object memory, whereas, the perirhinal cortex is involved in the accumulation of perceptual information during the initial exploration of novel objects (Cinalli et al., 2020; Stackman et al., 2016). We did not observe any association between the alEC volume and object recognition performance, contrary to our hypothesis and to previous research (Berron et al., 2018; Reagh et al., 2018; Reagh & Yassa, 2014).

We discovered that the AlEC volumes were associated with the ability to recognize which object belonged to which context in the object‐in‐context subtask. This result supports the notion that alEC might hold contextual information needed for processing at later stages in the HC (Knierim et al., 2014), corroborated by direct recordings in rodent's entorhinal cortices showing spatial tuning to objects in relation to visual landmarks and patterns of the environment (Deshmukh et al., 2012; Deshmukh & Knierim, 2011; Neunuebel et al., 2013). Our results are consistent with the recent fMRI findings in humans supporting the PMAT framework (Ranganath & Ritchey, 2012; Ritchey et al., 2015), and by extension to studies which found deficits in object processing implied by tau burden in the alEC (Berron et al., 2019; Maass et al., 2019). To our best knowledge, this is the first evidence for deficits in alEC functions that might go beyond the identity of the object and might extend to the relation between the object identity and environmental constituents.

The OLT task could be interpreted in terms of the posterior‐medial system of the PMAT framework owning its involvement in spatial contextual processing (Ritchey et al., 2015). Indeed, parahippocampal cortex, retrosplenial cortex, inferior parietal cortex and precuneus have been associated with the ability to retrieve object information within contexts, and with goal‐oriented behavior in navigation (Auger et al., 2012; Maguire, Burgess, et al., 1998; Marchette et al., 2014; Uncapher et al., 2006; Weniger et al., 2011). Parahippocampal cortex and retrosplenial cortex have been associated with object configuration and object in place memory (Bohbot et al., 2015; Yeung et al., 2019) with an ensemble of cells with place fields sensitive to environmental cues (Burwell & Hafeman, 2003; Jacob et al., 2017). Importantly, further evidence has suggested these areas are more critically involved in holding the temporal or ordinal aspects relative to the recollection (Hsieh et al., 2014; Hsieh & Ranganath, 2015), which is negligible in the OLT presented in the study as item presentation was pseudo‐randomized and the object order was not directly asked to the participants.

### 4.5 Limitations

There are some limitations in our tasks that are worth discussing. Each subtask presented a number of trials which is lower than the average number of trials in analogous tasks, as found in the literature, leading to a quantization of metrics and deviations from normality. However, our primary aim was to avoid long testing sessions to minimize fatigue in our participants, a typical concern when working with an aging population. We instead prioritized including an extensive tutorial to address the lack of virtual reality exposure in older subjects and to address the memory load required for retaining instructions. While removing the tutorial would minimize the time required to take the current test to only half an hour long, this would still be longer than the administration time of the standard neuropsychological tests. (Wang et al., 2018). To achieve the same goal, the recognition memory could be prompted at the same time as showing the object to the participant for the replacement. Another limitation is that the conclusions that can be drawn from our results are restricted by the small sample sizes of aMCI available with biomarkers and by the unrepresentatively high number of years in education in the older participant groups.

Each subtask represents an interesting investigating question to address using iVR and could be made into its own dedicated task. Separating the tasks will provide an alternative possibility to address the trial‐size limitation of each subtask and additionally provide the possibility to incorporate a stepwise systematic increase in either the number of objects or the number of the environments. The stepwise increase in the number of probed elements (objects and/or environments) would expand the range of task difficulty, and consequently the range of abilities being tested. Such a task would potentially offer greater classification accuracy given the greater scope to capture performance differences.

Our hypothesis required the manual segmentation of EC and BA35 whereas the whole HC was segmented using Freesurfer 6. Manual segmentation remains the gold standard in volumetric estimates (Schmidt et al., 2018) yet despite the Freesurfer's high test–retest reliability (Brown et al., 2020) and our manual inspection of the output, we cannot exclude the possibility that this methodological difference may have biased the results.

## 5 CONCLUSIONS

We present a novel VR OLT probing different aspects of EC and hippocampal function. This initial application to clinical cohorts has found that patients with mild cognitive impairment have selective deficit in object location memory. Consistent with the evolving understanding of the role of anterolateral EC in jointly representing spatial and contextual information, and the early pathological involvement of this region in AD, patients with prodromal AD (MCI with positive CSF biomarkers) were preferentially impaired on a test of object‐in‐context binding. These initial results highlight the potential value of EC‐dependent object‐location tasks in detection of AD in its earliest, preclinical, stages.

## CONFLICT OF INTEREST

The authors declare no conflict of interests.

## Supporting information


**Appendix S1** Supporting information

## Data Availability

The anonymized datasets used and/or analyzed in the current study are available after publication from the corresponding author on written request.
