# Supplementary material for: Assessing mild cognitive impairment using object‐location memory in immersive virtual environments
Source: Hippocampus. 2022 Aug 2;32(9):660–78. doi: 10.1002/hipo.23458 (PMC9543035; doi:10.1002/hipo.23458)
Supplement: Supplementary file 1 — Appendix S1 Supporting information [file HIPO-32-660-s001.docx]

# Supplementary Information

## Detection of Location Error Bindings

Location binding errors are assessed using the ‘nearest neighbor’ algorithms to detect which given response is the nearest to the veridical object locations regardless of the object identities (Pertzov et al., 2012). In the current study, considering the tracking limitation of the iVR system, objects could not be placed more than one meter apart from each other, resulting in a possible inflation of location binding errors if using the ‘nearest neighbor’ algorithm. To assess the location binding errors, we developed an in-house heuristic algorithm focusing on the geometry of each configuration of objects. The algorithm ensures that a location binding error was assessed controlling for how close the objects were in each configuration such as a binding error was more difficult to flag in configurations where objects were close rather than far between each other. The algorithm works as follows: two polygons were created using the vertices of the veridical object locations, Vr_i_ (i being the index in the configuration), and using the vertices of the subject response locations, Vs_i_ (i being the index of the configuration). A structural error, indicating the overlap between the two polygons was defined as the sum of the distances between each paired vertex. To assess the quality of each response polygon relative to the veridical polygon, a general 2D transform matrix was built to transform the vertices of the response polygon (Vs_i_) accounting for scaling, rotation and translation as follows

$$\vec{Vs}_{i}^{'}=T \vec{Vs}_{i}= S R T \vec{Vs}_{i}$$

where

$$S=\left[ \begin{matrix} k & 0 & 0 \\ 0 & k & 0 \\ 0 & 0 & 1 \end{matrix} \right], R= \left[ \begin{matrix} \cos\theta& \sin\theta& 0 \\ -\sin\theta& \cos\theta& 0 \\ 0 & 0 & 1 \end{matrix} \right], T= \left[ \begin{matrix} 1 & 0 & 0 \\ 0 & 1 & 0 \\ t_{x} & t_{y} & 1 \end{matrix} \right]$$

*S* is a scaling matrix, *R* is a rotation matrix, *T* is a translation matrix. Shearing was not allowed, as after the transformation, the geometry of the configuration created by the participant had to be preserved. A non-linear solver was then used to extract the optimal values of *k, ϑ, t_x_* and *t_y_* by minimizing the structural error, acting as a cost function for the solver.

$$C\left( k,\theta,t_{x},t_{y} \right)= \sum_{i=1}^{4} \sqrt{\left( \vec{Vs}_{i}^{'}- \vec{Vr}_{i} \right)^{T}(\vec{Vs}_{i}^{'}-\vec{Vr}_{i})}$$

*C(k,ϑ,t_x_,t_y_)* is the structural error distance calculated after a generic transformation of the participant response vertices indicating the difference between the response polygon and the veridical polygon. If the participant had placed the objects in proximity of their veridical location, the optimal cost function approached zero, indicating that each object could be returned close to its veridical location.

To detect the binding errors, the minimum cost function was subsequently calculated for all the response polygons obtained by either duplicating one response vertex index in the configuration - e.g., 1224 -- or after the permutation of two indices - e.g., 1324 - or after the permutations of three indices -- e.g., 4231. By ranking all the permutated cost functions, the algorithm finds the candidate for the binding error.

The final step of the algorithm controlled for false positives by including a check on the best candidates looking for a guess chance of placing the object in the wrong location. To assess the proximity of one candidate object over the location of another object controlling for the guess chance, we defined a “catchment” area centred on each veridical vertex location. The catchment area was defined as the mean error distance of the veridical vertex location to each binned point of the tracked area (0.1 meter bin) weighted over the mean sum of the absolute distances to the other veridical vertices locations in the configuration. With such definition, objects more isolated in the configuration had a smaller catchment area whereas objects closer to others had a relatively larger catchment area. A candidate binding error was considered valid if the replaced object exited the catchment area of its veridical vertex and entered the catchment area of another veridical vertex location. The algorithm has been run independently for each configuration and for each participant. An example of the detected errors and the catchment areas can be found in supplementary figure 1.


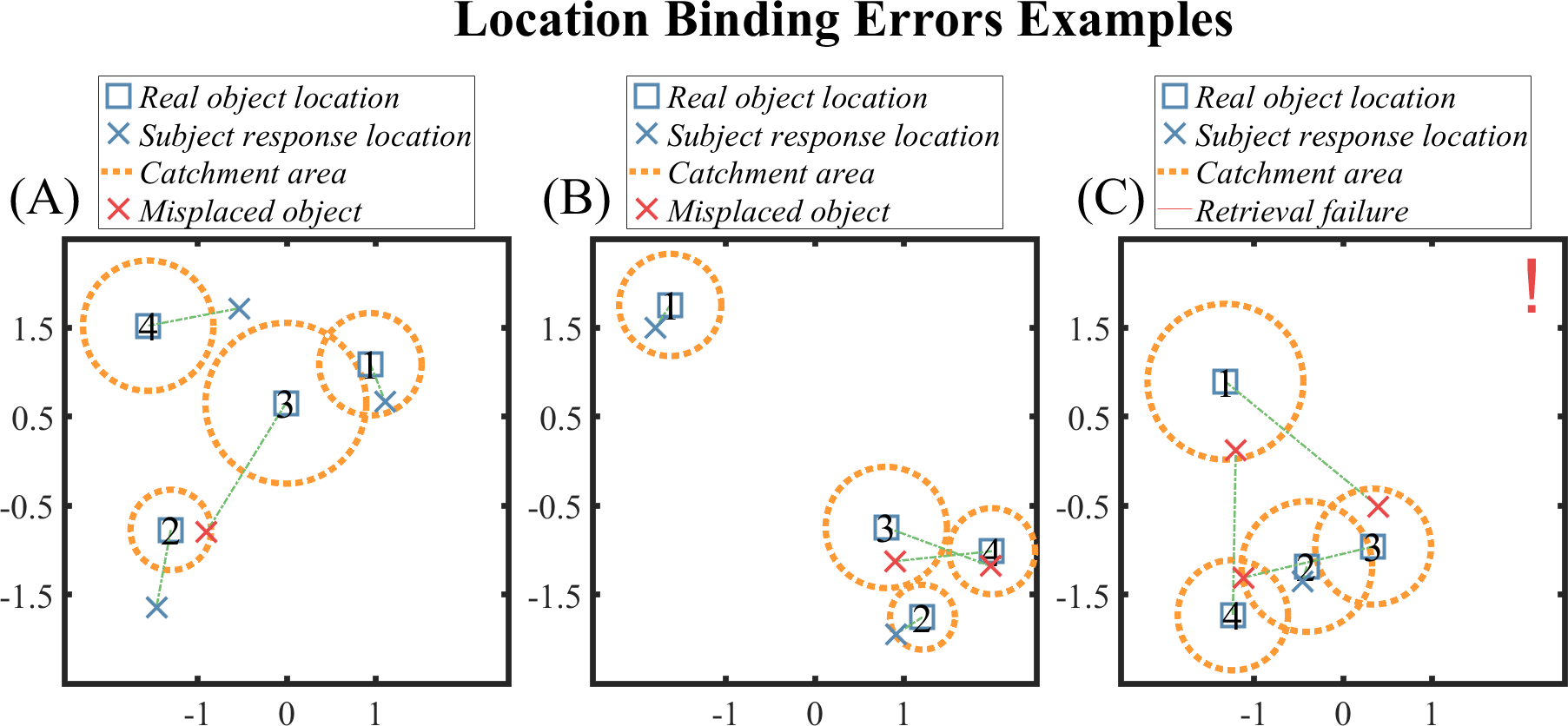


Supplementary figure 1: **Location Binding Error Examples**. Location binding error occurs when a cued object is set in the proximity of another object belonging to the same configuration. A custom algorithm detected location binding error using the geometric configuration of the set of objects (see supplementary methods for more information). In the picture, three examples for detected errors are shown. Retrieval failure occurred when participants misplaced three or more objects. In each plot, the real locations of the objects are reported with blue squares containing the index of the object within the configuration. Crosses indicate participant’s responses, with blue indicating valid responses and red indicating detected location binding errors. Green dashed line indicates the distance between the cued object and the participant’s response. The yellow circular dashed line indicates the ‘catchment’ area – a safe area where the response for a cued object was not considered erroneous in the location binding error algorithm.

## Object-in-context performance

Given the small sample size of the patient group tested and the data quantization, non-parametric analysis has been performed to assess the differences between the group distributions in the object-in-context subtask. A Kruskal-Wallis test did not reveal any differences in the distribution between young, older, and pooled MCI [χ2(2,97) = 6.05, p = 0.05]. A Mann–Whitney–Wilcoxon test revealed that the median in percentage accuracy in object-in-context performance was greater for the MCI+ (m = 67) than the MCI- (m = 56; U = 76, p = 0.046].
